# Dose- and Organ-Specific Dual Effects of MitoTempo in Paracetamol-Induced Hepatorenal Toxicity in Mice

**DOI:** 10.3390/biom16040556

**Published:** 2026-04-09

**Authors:** Hilmi Orhan, Kemal Atmaca, Berin Aladağ, Mustafa Kotmakçı

**Affiliations:** 1 Department of Pharmaceutical Toxicology, Faculty of Pharmacy, Ege University, 35040 Bornova-İzmir, Türkiye; 2 Department of Pharmaceutical Biotechnology, Faculty of Pharmacy, Ege University, 35040 Bornova-İzmir, Türkiye

**Keywords:** paracetamol, mitotempo, hepatotoxicity, nephrotoxicity, mitochondria, oxidative stress, mouse

## Abstract

Paracetamol (PAR) overdose is a major cause of drug-induced liver injury and is also associated with renal toxicity, both involving mitochondrial dysfunction and oxidative stress. This study investigated the dose- and organ-specific effects of the mitochondria-targeted antioxidant MitoTempo (MT) on PAR-induced hepatorenal toxicity in mice. Male C57BL/6J mice received a single toxic dose of PAR (600 mg/kg), either alone or combined with MT (20 or 40 mg/kg). Twenty-four hours after treatment, serum markers of liver and kidney injury were measured, and mitochondrial function was assessed in both organs. PAR administration caused severe liver injury and moderate renal dysfunction, accompanied by increased mitochondrial oxidative stress, glutathione imbalance, mitochondrial permeability transition pore opening, and disruption of electron transport chain (ETC) integrity. MT co-treatment attenuated several PAR-induced mitochondrial alterations in a dose- and tissue-dependent manner; however, MT did not consistently confer protection and, in some settings, exacerbated oxidative stress and bioenergetic dysfunction, particularly in the kidney. Notably, recovery of ETC protein levels by MT was not consistently associated with restoration of enzymatic activity. Overall, these findings demonstrate that MT exerts dual, dose- and organ-specific effects on PAR-induced mitochondrial dysfunction, highlighting that mitochondria-targeted antioxidants are not universally protective.

## 1. Introduction

Paracetamol (acetaminophen, PAR) overdose is a leading cause of acute liver failure in humans and may result in severe morbidity and mortality. The molecular mechanisms underlying PAR-induced hepatotoxicity have been extensively characterized. Following overdose, excessive formation of the reactive metabolite *N*-acetyl-*p*-benzoquinone imine (NAPQI) overwhelms the detoxification capacity of glutathione (GSH), leading to covalent binding of NAPQI to intracellular proteins, particularly within mitochondria. This initiates profound mitochondrial oxidative stress characterized by increased superoxide production from the electron transport chain (ETC). Superoxide reacts with nitric oxide to generate peroxynitrite, a highly reactive nitrogen species that induces oxidative damage to mitochondrial DNA and nitration of mitochondrial proteins, including mitochondrial superoxide dismutase (SOD2). Nitration-mediated inactivation of SOD2 further exacerbates mitochondrial oxidant stress through accumulation of hydrogen peroxide. The ensuing redox imbalance promotes activation and phosphorylation of c-Jun N-terminal kinase (JNK), which translocates to mitochondria and amplifies mitochondrial dysfunction. Under conditions of severe oxidative stress, disruption of mitochondrial membrane integrity triggers opening of the mitochondrial permeability transition pore (mPTP), resulting in the release of endonucleases, cytochrome *c*, and apoptosis-inducing factor (AIF), nuclear DNA fragmentation, and ultimately a regulated form of necrotic cell death that underlies PAR-induced liver injury [1,2,3,4]. As emphasized previously, mitochondrial dysfunction and oxidative stress represent the two central and inseparable hallmarks of PAR-induced hepatotoxicity [1]. Although liver injury is the primary clinical concern following PAR overdose, renal toxicity is also well documented. Acute kidney injury may develop with or without concomitant hepatotoxicity, yet often receives less clinical attention [5,6,7,8,9]. Importantly, *N*-acetylcysteine (NAC), the standard antidote for PAR-induced hepatotoxicity, is ineffective in preventing nephrotoxicity [10]. While NAC restores hepatic GSH levels, renal GSH depletion is minimal, and inhibition of CYP2E1-mediated NAPQI formation—rather than antioxidant replenishment—appears critical for renal protection [11]. Consistent with this notion, our previous work demonstrated that, unlike in the liver, PAR-induced renal injury occurs in the absence of mitochondrial protein alkylation, JNK activation, or JNK translocation to mitochondria, despite clear evidence of kidney damage [12]. These findings indicate that the downstream mechanisms of PAR-induced nephrotoxicity differ fundamentally from those driving hepatotoxicity. Given the central role of mitochondria-derived oxidative stress in PAR-induced liver injury, mitochondria-targeted antioxidants have emerged as promising experimental tools to dissect mitochondrial redox mechanisms and explore potential therapeutic strategies. Among these compounds, MT—a piperidine nitroxide conjugated to a triphenylphosphonium cation—selectively accumulates within mitochondria driven by the mitochondrial membrane potential and primarily functions as a mitochondrial superoxide scavenger. Previous studies have demonstrated that MT confers significant protection against PAR-induced hepatotoxicity by attenuating mitochondrial superoxide formation, limiting peroxynitrite generation, preserving ETC integrity, and reducing hepatocellular necrosis [13,14]. However, these same studies also revealed that MT’s effects are not universally beneficial. In particular, delayed phases of injury and limited secondary apoptosis have been observed, highlighting that modulation of mitochondrial redox signaling may produce context-dependent outcomes influenced by dose, timing, and injury phase [13]. Further support for the complexity of mitochondria-targeted antioxidant actions comes from studies using related compounds such as MitoQ, which have shown alterations in mitochondrial membrane lipid composition, including increased cardiolipin content, with potential consequences for ETC organization and bioenergetic function [15]. Collectively, these findings indicate that mitochondria-targeted antioxidants exert biological effects that extend beyond simple radical scavenging and may differentially influence mitochondrial structure, function, and redox balance depending on tissue context. Despite the established role of mitochondrial oxidative stress in PAR-induced hepatotoxicity, the contribution of mitochondrial redox alterations to PAR-induced nephrotoxicity remains incompletely understood. This gap in knowledge is particularly relevant given the absence of canonical mitochondrial death signaling pathways in the kidney and raises the possibility that interventions effective in the liver may not translate directly to renal protection. Accordingly, a systematic, organ-specific evaluation of mitochondrial oxidative stress and function in response to MT during PAR intoxication is warranted. In this context, we hypothesized that paracetamol-induced mitochondrial dysfunction exhibits distinct organ- and dose-specific characteristics and that modulation by the mitochondria-targeted antioxidant MT differs between liver and kidney. The novelty of the present study lies in the integrated, organ-specific assessment of mitochondrial redox status, bioenergetic function, and electron transport chain regulation at the mRNA, protein, and activity levels within the same experimental framework. In the present study, we investigated in detail the effects of MT administered alone or at two different doses in combination with a single toxic dose of PAR on mitochondrial oxidative stress, ETC integrity, and functional outcomes in mouse liver and kidney. We comprehensively assessed mitochondrial reactive oxygen species (ROS) production, lipid peroxidation, the GSSG/GSH ratio, oxidative DNA damage, mitochondrial membrane potential, and mPTP induction. In parallel, we evaluated the expression, protein abundance, and enzymatic activity of ETC complexes I–IV, including analysis of individual Complex I subunits. All parameters were measured simultaneously in liver and kidney tissues to allow direct organ-specific comparison. Through this integrated approach, we aimed to elucidate whether modulation of mitochondrial redox balance by MT exerts uniform protective effects or instead produces dose- and tissue-specific outcomes that may explain differential susceptibility to PAR-induced hepatorenal toxicity.

## 2. Materials and Methods

### 2.1. Chemicals and Reagents

Paracetamol (acetaminophen, PAR; analytical grade) was kindly provided by Abdi İbrahim Pharmaceutical Company (Istanbul, Türkiye). MT (2-(2,2,6,6-tetramethylpiperidin-1-oxyl-4-ylamino)-2-oxoethyl triphenylphosphonium chloride) was purchased from Merck (SML0737; Sigma–Aldrich) (St. Louis, MO, USA). MitoSOX™ Red mitochondrial superoxide indicator (M36008), JC-1 mitochondrial membrane potential assay dye (T3168), calcein-AM, and CoCl_2_ were obtained from Thermo Fisher Scientific (Waltham, MA, USA). Thiobarbituric acid (TBA), trichloroacetic acid (TCA), pyrogallol, NADH, potassium phosphate (KP) buffer components, cytochrome c (oxidized and reduced forms), decylubiquinol, Coenzyme Q1 (CoQ1), Tween-20, potassium cyanide (KCN), Tris-HCl, EDTA, sucrose, mannitol, bovine serum albumin (BSA), and all other general laboratory chemicals were purchased from Merck (Sigma–Aldrich) unless otherwise stated. Ellman’s reagent (5,5′-dithiobis-(2-nitrobenzoic acid); DTNB) and glutathione reductase were obtained from Sigma–Aldrich. The 8-hydroxy-2′-deoxyguanosine (8-OHdG) ELISA Kit was purchased from Elabscience Biotechnology Inc. (E-EL-0028; Houston, TX, USA). The Nucleoside Digestion Mix was obtained from New England Biolabs (Ipswich, MA, USA). For nuclear DNA isolation, the Thermo Scientific™ (Waltham, MA, USA) GeneJET Genomic DNA Purification Kit was used (K0722). Total RNA Isolation Kit (74104), and cDNA Synthesis Kit (05311) were obtained from Qiagen (Hilden, Germany). Quantitative PCR was carried out using RT^2^ SYBR^®^ Green Mastermix (Qiagen). Primary antibodies against mitochondrial ETC complexes I–IV were obtained from Abcam (ab110242, ab14714, ab14745, and ab14705, respectively; Cambridge, UK). Horseradish peroxidase (HRP)-conjugated secondary antibodies (7074) and enhanced chemiluminescence (ECL) detection reagents were purchased from Cell Signaling Technology (32209; Danvers, MA, USA). Bradford reagent for protein quantification was obtained from Bio-Rad Laboratories (Hercules, CA, USA). Biochemical parameters (ALT, AST, ALP, urea, and BUN) were measured using commercially available kits compatible with the Randox Rx Daytona Plus automated biochemistry analyzer (AL8304, AS8306, AP8302, and UR8334, respectively; Randox Laboratories, Crumlin, UK). Unless otherwise specified, all reagents were of analytical grade, and all solutions were prepared using ultrapure distilled water.

### 2.2. Animals and Experimental Design

All animal procedures were approved by the Izmir Biomedicine and Genome Center (IBG) Ethics Committee (protocol no. 2021-009). Male C57BL/6J mice (8–12 weeks old, 20–30 g) were obtained from the IBG Vivarium and acclimated for one week under standard laboratory conditions with free access to food and water. Following acclimatization, animals were randomly assigned to six experimental groups (*n* = 6 per group): control, PAR, MitoTempo 20 (MT20), MitoTempo 40 (MT40), PAR + MT20, and PAR + MT40. Mice were fasted for 15 h prior to drug administration. PAR was administered at a single toxic dose of 600 mg/kg body weight, while MT was administered at doses of 20 or 40 mg/kg body weight. All treatments were delivered via intraperitoneal injection. MT was administered either alone or concomitantly with PAR according to group allocation. Twenty-four hours after treatment, blood samples were collected by intracardiac puncture under isoflurane anesthesia for biochemical analyses. Animals were then euthanized by desanguination, and liver and kidney tissues were rapidly excised, rinsed in ice-cold saline, and processed immediately or stored at −80 °C until further analysis. All biochemical measurements were performed by investigators blinded to the experimental group allocation. All samples were processed and analyzed together for each parameter in a single experimental run to avoid batch effects.

### 2.3. Biochemical Assays

Blood samples were centrifuged at 16,000× *g* for 5 min at 4 °C. The resulting serum was transferred to clean tubes and stored at −20 °C until analysis. Serum alanine aminotransferase (ALT), aspartate aminotransferase (AST), and alkaline phosphatase (ALP) activities were measured as indicators of hepatocellular injury, while blood urea nitrogen (BUN) and urea levels were assessed as markers of renal function. All biochemical parameters were determined using appropriate commercial kits on a Randox Rx Daytona Plus automated biochemistry analyzer.

### 2.4. Preparation of Mitochondrial Fractions

Liver and kidney tissues were weighed and homogenized in five volumes of ice-cold mitochondrial isolation buffer (20 mM HEPES, 70 mM sucrose, 220 mM mannitol, 2 mM EDTA, 0.5 mg/mL BSA, pH 7.5) using a glass Potter homogenizer [16,17]. Homogenates were centrifuged at 800× *g* for 10 min at 4 °C to remove cellular debris and nuclei. The resulting supernatants were centrifuged at 8000× *g* for 15 min at 4 °C to obtain mitochondrial pellets. Pellets were washed three times with ice-cold PBS to remove cytosolic contaminants and subsequently homogenized in PBS using an ultrasonicator (3 × 10 s pulses at 30 kHz) on ice. Final mitochondrial homogenates were stored at −80 °C until analysis. Protein concentrations were determined using the Bradford assay. Briefly, 250 μL of Bradford reagent was mixed with 5 μL of mitochondrial homogenate, incubated for 10 min at room temperature in the dark, and absorbance was measured at 595 nm using Varioskan Flash multimode plate reader (Thermo Fisher Scientific, Vantaa, FI, USA). Protein concentrations were calculated using a bovine serum albumin (BSA) standard curve.

### 2.5. Measurement of Mitochondrial Reactive Oxygen Species

Mitochondrial ROS production was assessed using freshly isolated, intact (non-homogenized) mitochondria. Mitochondrial suspensions were transferred to black 96-well plates, and PBS containing 5 μM MitoSOX™ Red(Thermo Fisher Scientific, Waltham, MA, USA) was added. After incubation at 37 °C for 30 min, fluorescence intensity was measured at excitation/emission wavelengths of 510/580 nm using Varioskan Flash multimode plate reader (Thermo Fisher Scientific, Waltham, MA, USA) [18].

### 2.6. Measurement of Mitochondrial Lipid Peroxidation

Mitochondrial lipid peroxidation was quantified by measuring malondialdehyde (MDA) levels using the thiobarbituric acid-reactive substances assay [19]. Briefly, 100 μL of mitochondrial homogenate was mixed with 200 μL of TBA reagent (15% *w*/*v* trichloroacetic acid, 0.375% *w*/*v* TBA, and 0.25 N HCl) and incubated at 100 °C for 15 min. After cooling to room temperature, samples were centrifuged at 1000× *g* for 10 min. Supernatants were transferred to a 96-well plate, and absorbance was measured at 535 nm. Results were normalized to protein content and expressed as nmol MDA/mg protein.

### 2.7. Quantification of Mitochondrial GSSG/GSH Ratio

Mitochondrial glutathione levels were determined spectrophotometrically using Ellman’s reagent. For reduced glutathione (GSH) measurement, 150 μL of EDTA buffer (pH 8.2) and 10 μL of DTNB were added to 50 μL of mitochondrial homogenate and incubated at 37 °C for 30 min. Absorbance was measured at 412 nm. For oxidized glutathione (GSSG) determination, samples were treated with glutathione reductase to reduce GSSG to GSH, and total glutathione was measured using the same protocol. GSH and GSSG concentrations were calculated from standard curves of each compound, and results were expressed as the GSSG/GSH ratio [20].

### 2.8. Mitochondrial Superoxide Dismutase Activity Assay

SOD2 activity was measured by monitoring the inhibition of pyrogallol autooxidation following modification [21,22]. Briefly, 150 μL of assay buffer (50 mM Tris-HCl, 1 mM EDTA, pH 8.2) was mixed with 10 μL of mitochondrial homogenate. The reaction was initiated by the addition of 2 mM pyrogallol (final), and absorbance was recorded at 420 nm every 30 s for 450 s. Enzyme activity was expressed as units per milligram of protein (U/mg protein).

### 2.9. Measurement of 8-OHdG in Mitochondrial and Nuclear DNA

Mitochondrial and nuclear DNA were isolated separately. Mitochondrial DNA was extracted according to the protocol described by Kauppila et al. [23], while nuclear DNA was isolated using a commercial genomic DNA purification kit. DNA concentration and purity were assessed spectrophotometrically. For enzymatic hydrolysis, 1 μg of DNA was digested using a nucleoside digestion mix to release 8-OHdG. Quantification was performed using a competitive ELISA kit according to the manufacturer’s instructions. Results were expressed as ng 8-OHdG per ng DNA.

### 2.10. Assessment of Mitochondrial Membrane Potential

Mitochondrial membrane potential (Δψm) was assessed using the JC-1 fluorescent dye. Isolated mitochondria were transferred to black 96-well plates and incubated with PBS containing 20 μM JC-1 at 37 °C for 10 min. Fluorescence was measured at excitation/emission wavelengths of 485/535 nm (monomeric form) and 535/595 nm (aggregated form). Results were expressed as the ratio of monomeric to aggregated JC-1 fluorescence [24].

### 2.11. Evaluation of Mitochondrial Permeability Transition Pore Opening

mPTP opening was evaluated using the calcein–cobalt quenching assay. Isolated mitochondria were incubated with PBS containing 1 μM calcein-AM and 1 mM CoCl_2_ at 37 °C for 15 min. Fluorescence intensity was measured at excitation/emission wavelengths of 488/509 nm [25].

### 2.12. Gene Expression Analysis of Electron Transport Chain Components

Total RNA was isolated from liver and kidney tissues, and RNA concentration and purity were determined spectrophotometrically [26]. Complementary DNA (cDNA) was synthesized according to the manufacturer’s protocol. Quantitative real-time PCR was performed using SYBR Green chemistry. The primers utilized for the PCR analysis of mitochondrial ETC complexes I, II, III, and IV, as well as the 16S rRNA reference gene, are listed in Table 1.

Each reaction contained 1 μL cDNA, 1 μL primer mix (10 μM), 10.5 μL RNase-free water, and 12.5 μL SYBR Green Master Mix. Amplification was carried out using a LightCycler 480 system (Roche Diagnostics, Mannheim, Germany) under the following conditions: initial denaturation at 95 °C for 3 min, followed by 45 cycles of 95 °C for 15 s, 60 °C for 30 s, and 72 °C for 1 min. Relative gene expression was calculated using the 2^−ΔΔCt^ method, with mitochondrial 16S rRNA used as the reference gene. For each ETC complex, the stability of mitochondrial 16S rRNA expression was evaluated independently across all treatment groups. No significant variation in reference gene Ct values was observed in liver or kidney tissues, confirming its suitability for normalization under the present experimental conditions.

### 2.13. Quantification of ETC Complex Proteins by Western Blotting

Western blot analysis was performed to determine the protein levels of ETC complexes. For this purpose, 5 μg of mitochondrial homogenate was loaded onto a 12% polyacrylamide gel, and electrophoresis was conducted in running buffer (25 mM Tris, 192 mM Glycine, 0.1% SDS) for approximately 2 h until the protein bands were separated. Following electrophoresis, the proteins were transferred to a PVDF membrane in transfer buffer (20% *w*/*v* methanol, 0.025% *w*/*v* SDS, 48 mM Tris, 39 mM glycine) at 4 °C for 2 h at 100 V. The membranes were then blocked with 5% non-fat milk powder at room temperature for 1 h and incubated overnight at 4 °C with primary antibodies on a shaker. After washing the membranes at least three times with TBS-T buffer, they were incubated with secondary antibodies for 1 h at room temperature. Finally, the membranes were washed again at least three times with TBS-T buffer, and the bands were visualized using a Vilber Lourmat Fusion FX7 imaging system (Paris, France) with an ECL detection kit. Band intensities were analyzed using ImageJ software (version 1.54g, National Institutes of Health, Bethesda, MD, USA).

### 2.14. Measurement of Enzymatic Activities of ETC Complexes

All measurements were performed as previously described by Brischigliaro et al. [27]. To assess Complex I activity, 186 μL of reaction mixture (50 mM KP buffer, pH 7.5, 3 mg/mL BSA, 300 μM KCN, 100 μM NADH) was added to 10 μL of mitochondrial homogenate. The reaction was initiated by adding 4 μL of 4 mM CoQ1, and the decrease in NADH absorbance at 340 nm was measured for 2 min. Activity was expressed as nmol NADH/min/mg protein. To measure Complex II activity, 196 μL of reaction mixture (25 mM KP buffer, pH 7.5, 1 mg/mL BSA, 300 μM KCN, 20 mM succinate, 75 μM DCPIP) was added to 2 μL of mitochondrial homogenate and incubated at room temperature for 10 min. The reaction was started by adding 2 μL of 4 mM CoQ1, and the decrease in DCPIP absorbance at 600 nm was measured for 2 min. Activity was expressed as nmol DCPIP/min/mg protein. For Complex III activity, 196 μL of reaction mixture (25 mM KP buffer, pH 7.5, 100 μM EDTA, 500 μM KCN, 0.025% Tween-20, 75 μM oxidized cytochrome c) was added to 2 μL of mitochondrial homogenate. The reaction was initiated by adding 2 μL of 10 mM decylubiquinol, and the absorbance decrease in cytochrome c at 550 nm was recorded for 2 min. Activity was expressed as nmol CytC/min/mg protein. To measure Complex IV activity, 198 μL of reaction mixture (50 mM KP buffer, pH 7.0, 75 μM reduced cytochrome c, 500 μM KCN) was added to 2 μL of mitochondrial homogenate. The decrease in cytochrome c absorbance at 550 nm was monitored for 2 min. Activity was expressed as nmol CytC/min/mg protein. Potassium cyanide (KCN) was obtained through authorized institutional procurement, handled by trained personnel under appropriate safety conditions in a certified chemical fume hood, and disposed of in accordance with institutional and regulatory hazardous waste management procedures.

### 2.15. Statistical Analysis

All statistical analyses were performed using GraphPad Prism 9 software. Data distribution was assessed with the Shapiro–Wilk normality test. For multiple comparisons, Tukey’s post hoc test was applied following parametric ANOVA, while Dunn’s test was used for non-parametric Kruskal–Wallis analyses. The Student’s t-test was used for two-groups comparisons. Results were expressed as mean ± SEM. Statistical significance was defined at *p* < 0.05, and different levels of significance (*p* < 0.05, *p* < 0.01, *p* < 0.001, and *p* < 0.0001) were denoted by numerical symbols as specified in the figure legends.

## 3. Results

### 3.1. Effects of Paracetamol and MT on Serum Markers of Hepatic and Renal Injury

**Figure 1:**
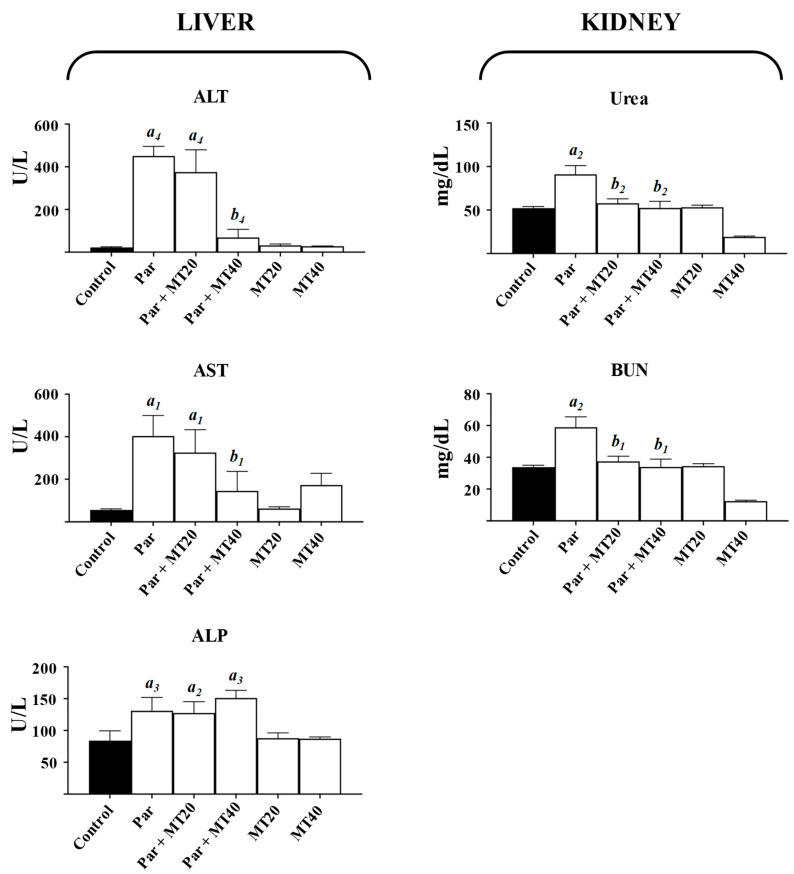
Serum markers of liver and kidney injury in control and treated mice. Serum activities of alanine aminotransferase, aspartate aminotransferase, and alkaline phosphatase were measured as indicators of hepatocellular injury, while urea and blood urea nitrogen levels were assessed as markers of renal dysfunction. PAR, paracetamol-treated group; PAR + MT20, paracetamol plus MT (20 mg/kg); PAR + MT40, paracetamol plus MT (40 mg/kg); MT20, MT (20 mg/kg); MT40, MT (40 mg/kg). Data are presented as mean ± SEM. a, vs. control; b, vs. paracetamol. Numbers 1–4 indicate statistical significance levels of *p* < 0.05, *p* < 0.01, *p* < 0.001, and *p* < 0.0001, respectively.

Hepatocellular injury results in the release of cytosolic enzymes into the circulation due to loss of plasma membrane integrity. Accordingly, liver injury was assessed by measuring serum alanine aminotransferase (ALT), aspartate aminotransferase (AST), and alkaline phosphatase (ALP) activities 24 h after PAR and/or MT administration (Figure 1).

Serum ALT activity increased markedly following PAR treatment, showing a 19.24-fold elevation compared with control animals. A similarly pronounced increase was observed in the PAR + MT20 group (15.85-fold). Co-treatment with MT20 resulted in a modest, non-significant reduction in ALT activity relative to PAR alone, whereas co-treatment with MT40 significantly reduced ALT activity by 1.85-fold compared with the PAR group. Administration of MT alone did not significantly affect ALT activity compared to control. Serum AST activity increased by 6.25-fold in the PAR group. Co-treatment with MT20 resulted in a non-significant reduction relative to PAR alone, whereas MT40 co-treatment significantly reduced AST activity by 1.64-fold compared with the PAR group. MT administered alone did not significantly alter AST activity. ALP activity increased significantly in the PAR (1.56-fold) group compared with control. Neither MT treatment alone at two doses nor co-treatment with PAR significantly altered ALP activity relative to control or PAR alone.

Renal dysfunction was assessed by measuring serum urea and blood urea nitrogen (BUN) levels (Figure 1). Serum urea levels increased significantly by 1.74-fold in the PAR group compared with controls. Co-treatment with MT20 and MT40 significantly reduced urea levels by 0.37-fold and 0.32-fold, respectively, relative to PAR alone, whereas no significant changes were observed in the other treatment groups. Similarly, BUN levels increased significantly by 1.74-fold in the PAR group, and this increase was significantly attenuated by MT20 and M40 co-treatment.

### 3.2. Effects of PAR and MT on Mitochondrial Oxidative Stress Parameters

**Figure 2:**
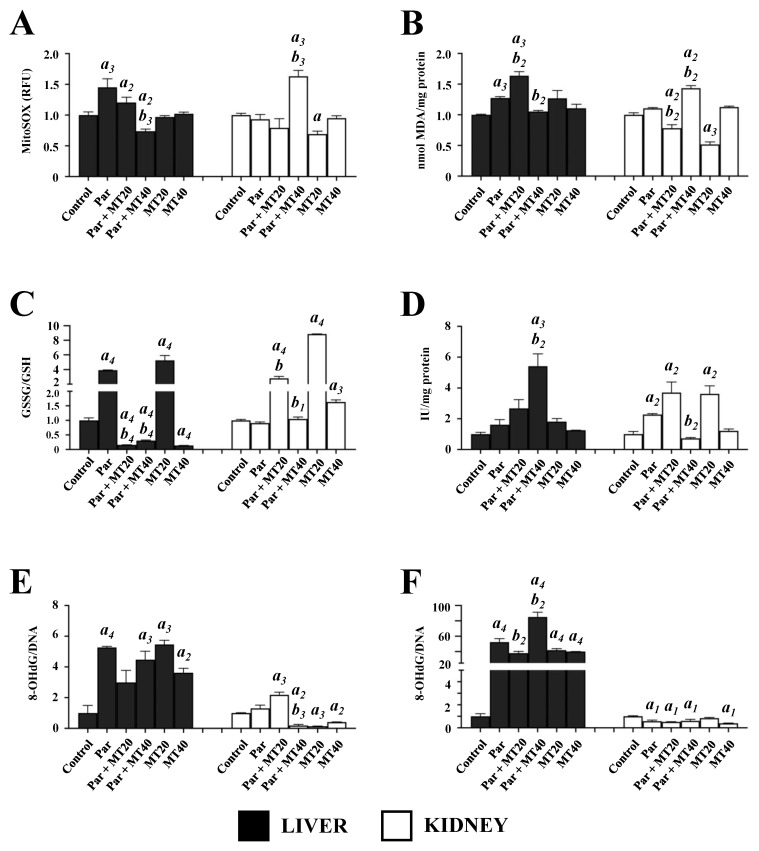
Effects of paracetamol and mitotempo on mitochondrial and nuclear oxidative stress parameters in mouse liver and kidney. Mitochondrial reactive oxygen species production (**A**), lipid peroxidation (**B**), glutathione redox status (**C**), and superoxide dismutase 2 activity (**D**) were assessed as indicators of mitochondrial oxidative stress. Oxidative DNA damage was evaluated by measuring 8-OHdG levels in mitochondrial (**E**) and nuclear DNA fractions (**F**). PAR, paracetamol-treated group; PAR + MT20, paracetamol plus MT (20 mg/kg); PAR + MT40, paracetamol plus MT (40 mg/kg); MT20, MT (20 mg/kg); MT40, MT (40 mg/kg). Data are presented as mean ± SEM. a, vs. control; b, vs. paracetamol. Numbers 1–4 indicate statistical significance levels of *p* < 0.05, *p* < 0.01, *p* < 0.001, and *p* < 0.0001, respectively.

Given the established role of mitochondrial reactive oxygen and nitrogen species in PAR-induced toxicity, multiple parameters of mitochondrial oxidative stress were assessed in isolated liver and kidney mitochondria (Figure 2). Mitochondrial ROS production was evaluated using the mitochondria-specific probe MitoSOX. In liver mitochondria, ROS levels increased significantly in the PAR (1.45-fold) and PAR + MT20 (1.20-fold) groups compared with controls. In contrast, ROS levels were significantly reduced in the PAR + MT40 group (0.74-fold vs. control) and decreased by approximately 49% compared with PAR alone. In kidney mitochondria, ROS levels increased significantly only in the PAR + MT40 group (1.63-fold vs. control), whereas a significant reduction (31%) was observed in the MT20 group. Compared with PAR alone, a significant increase in ROS levels was detected exclusively in the PAR + MT40 group. Mitochondrial lipid peroxidation was assessed by measuring MDA levels. In liver mitochondria, MDA levels increased significantly in the PAR (1.27-fold) and PAR + MT20 (1.64-fold) groups compared with controls. Relative to PAR alone, MDA levels increased modestly in the PAR + MT20 group but decreased significantly (17%) in the PAR + MT40 group. In kidney mitochondria, MDA levels were not significantly altered by PAR alone. However, significant reductions were observed in the PAR + MT20 (22%) and MT20 (48%) groups compared with controls. Relative to PAR alone, MDA levels decreased by 30% in the PAR + MT20 group but increased by 29% in the PAR + MT40 group.

Mitochondrial glutathione redox status was evaluated by determining the GSSG/GSH ratio. In liver mitochondria, the GSSG/GSH ratio increased markedly in the PAR (2.92-fold) and MT20 (4.24-fold) groups compared with controls. In contrast, significant reductions were observed in the PAR + MT20 (15%), PAR + MT40 (31%), and MT40 (14%) groups. Relative to PAR alone, both MT20 and MT40 co-treatment significantly reduced the GSSG/GSH ratio. In kidney mitochondria, no significant change was observed following PAR treatment alone. However, significant increases were detected in the PAR + MT20 (1.8-fold), MT20 (7.86-fold), and MT40 (1.63-fold) groups. Compared with PAR alone, the GSSG/GSH ratio increased significantly in both PAR + MT20 and PAR + MT40 groups. SOD2 activity was assessed in isolated mitochondria. In liver mitochondria, SOD2 activity increased significantly only in the PAR + MT40 group, showing a 4.42-fold increase compared with controls and a 2.37-fold increase relative to PAR alone. In kidney mitochondria, SOD2 activity increased significantly in the PAR + MT20 (2.71-fold) and MT20 (2.62-fold) groups compared with controls, whereas a significant decrease (32%) relative to PAR alone was observed exclusively in the PAR + MT40 group. Oxidative DNA damage was evaluated by measuring 8-OHdG levels in mitochondrial and nuclear DNA fractions. In liver mitochondrial DNA, 8-OHdG levels increased significantly in the PAR (4.27-fold), PAR + MT40 (3.37-fold), MT20 (4.46-fold), and MT40 (2.62-fold) groups compared with controls, with no significant differences relative to PAR alone. In kidney mitochondrial DNA, 8-OHdG levels increased modestly in the PAR + MT20 group but decreased significantly in the PAR + MT40, MT20, and MT40 groups compared with controls. Relative to PAR alone, a significant reduction was observed only in the PAR + MT40 group.

### 3.3. Effects of PAR and MT on Mitochondrial Permeability and Membrane Potential

**Figure 3:**
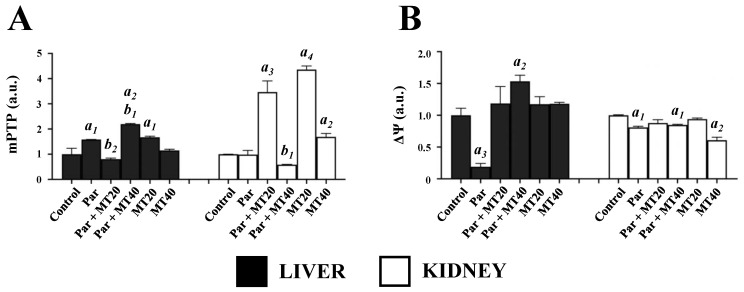
Effects of paracetamol and mitotempo on mitochondrial permeability transition pore opening (**A**) and mitochondrial membrane potential (**B**) in mouse liver and kidney. mPTP opening and MMP were assessed in isolated mitochondria following paracetamol and/or mitotempo treatment. PAR, paracetamol-treated group; PAR + MT20, paracetamol plus MT (20 mg/kg); PAR + MT40, paracetamol plus MT (40 mg/kg); MT20, MT (20 mg/kg); MT40, MT (40 mg/kg). Data are presented as mean ± SEM. a, vs. control; b, vs. paracetamol. Numbers 1–4 indicate statistical significance levels of *p* < 0.05, *p* < 0.01, *p* < 0.001, and *p* < 0.0001, respectively.

To assess mitochondrial integrity, mPTP opening and mitochondrial membrane potential (MMP) were evaluated in isolated liver and kidney mitochondria (Figure 3).

In liver mitochondria, mPTP opening increased significantly in the PAR (1.58-fold), PAR + MT40 (2.17-fold), and MT20 (1.67-fold) groups compared with controls. Relative to PAR alone, mPTP opening was significantly reduced by 51% in the PAR + MT20 group but increased by 1.39-fold in the PAR + MT40 group.

In kidney mitochondria, mPTP opening increased significantly in the PAR + MT20 (2.46-fold), MT20 (3.36-fold), and MT40 (1.69-fold) groups compared with controls. Relative to PAR alone, a marked increase was observed in the PAR + MT20 group, whereas a significant reduction (40%) was detected in the PAR + MT40 group. PAR treatment resulted in a pronounced decrease in MMP in liver mitochondria. This decrease was significantly reversed in the PAR + MT40 group, whereas no significant differences were observed in the PAR + MT20, MT20, or MT40 groups relative to controls. In kidney mitochondria, PAR induced only a modest reduction in MMP (~15%), which was prevented by MT20 co-treatment but not by MT40. Notably, MT40 administered alone significantly reduced kidney mitochondrial MMP by approximately 40% compared with controls.

### 3.4. Effects of PAR and MT on Mitochondrial Electron Transport Chain Complexes

The effects of PAR and MT on ETC components were evaluated by analyzing protein abundance by Western blotting (representative blots are shown in the main figures; original uncropped blots are provided in Supplementary Figures S1–S10), together with mRNA expression and enzymatic activities of ETC complexes I–IV in liver and kidney mitochondria.

#### 3.4.1. Complex I

**Figure 4:**
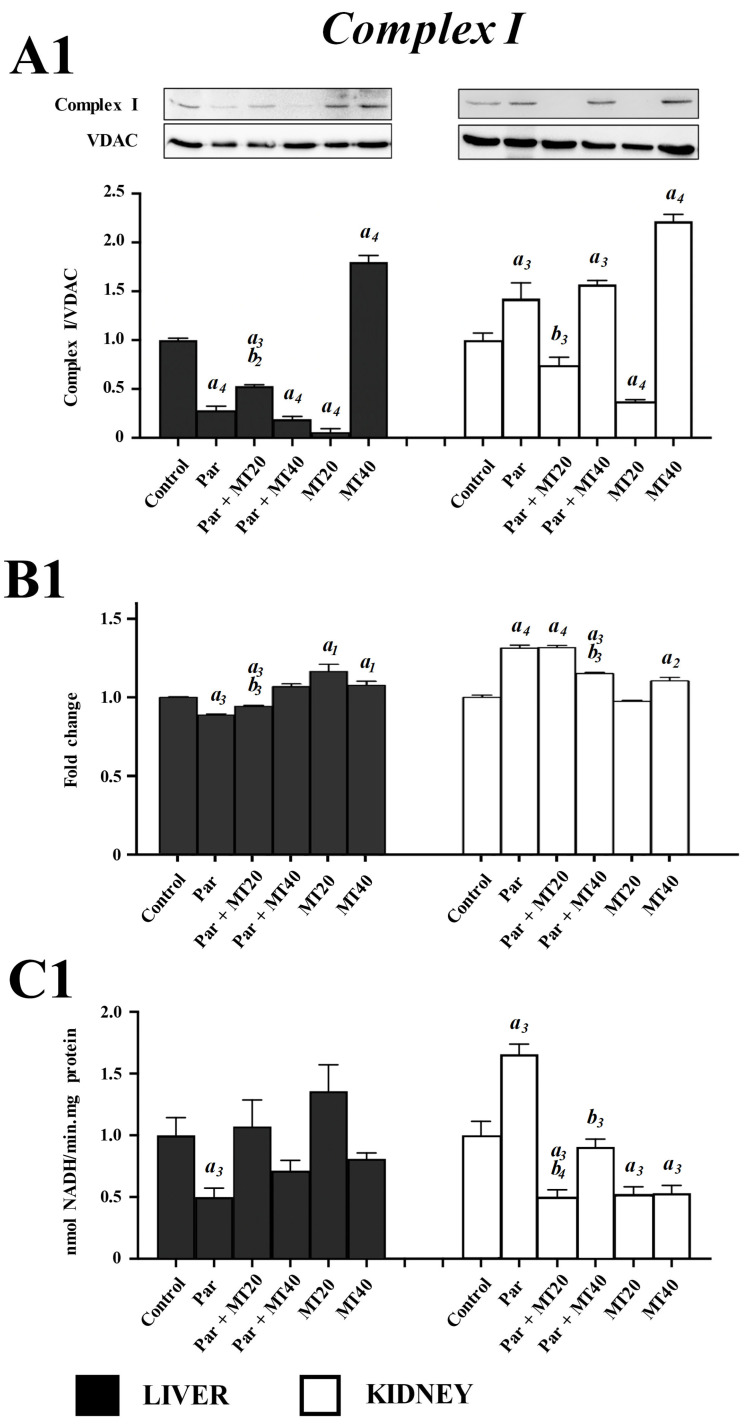

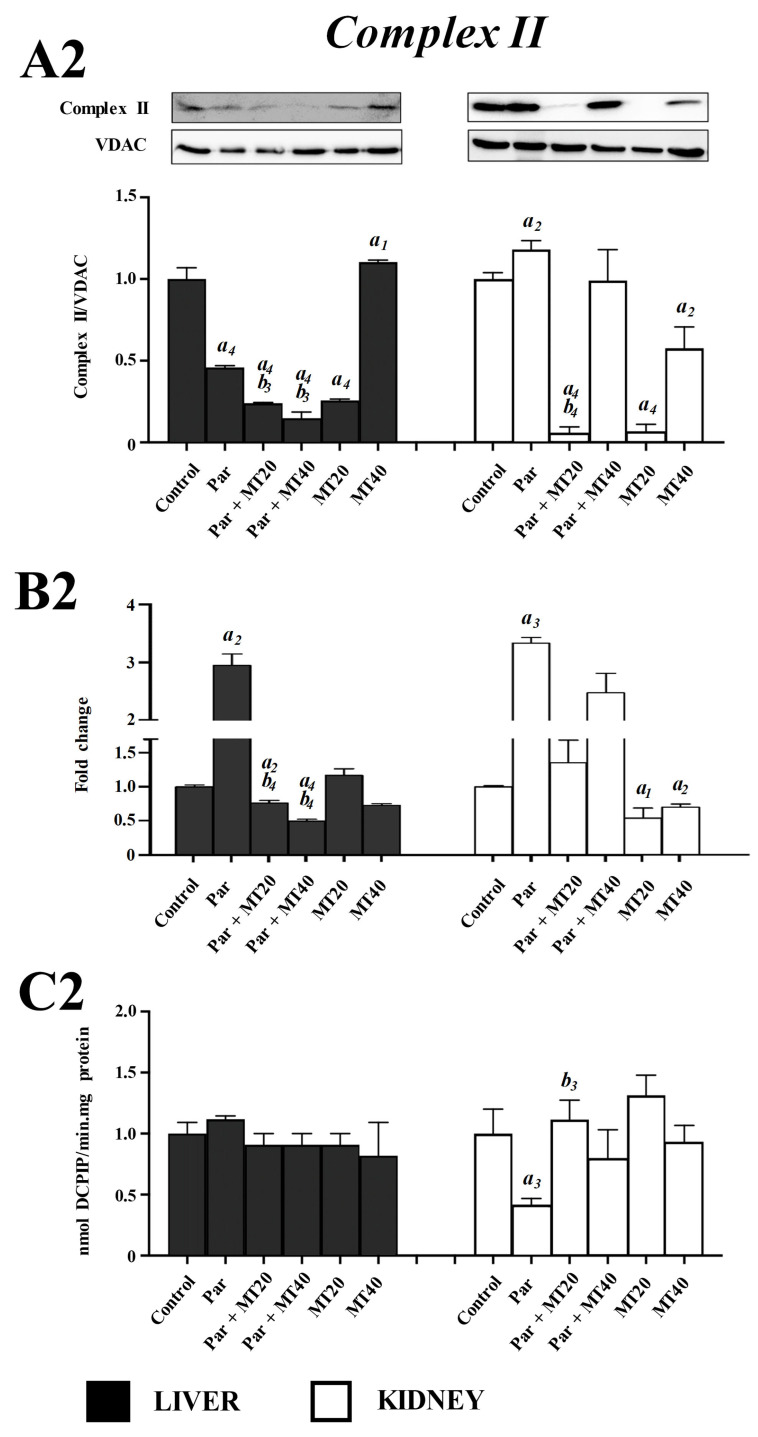

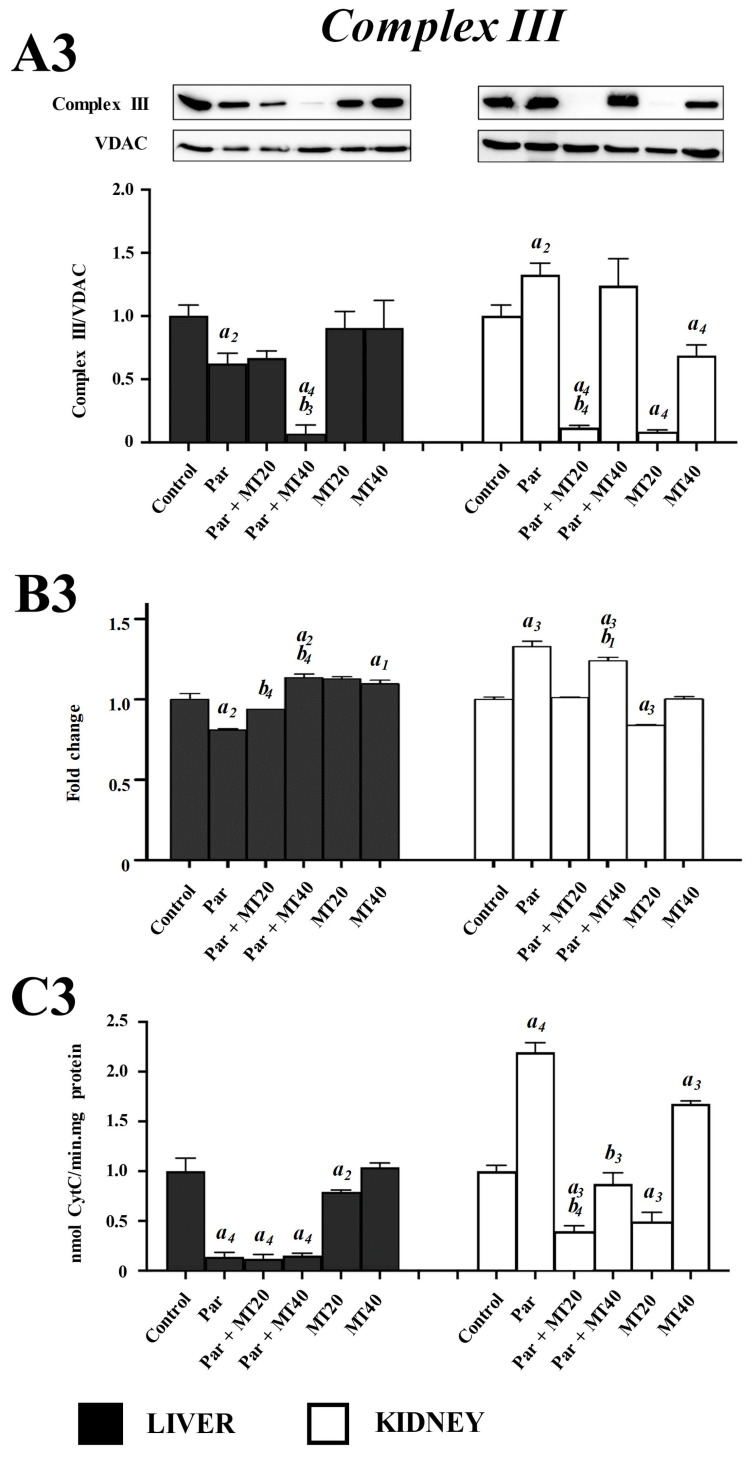

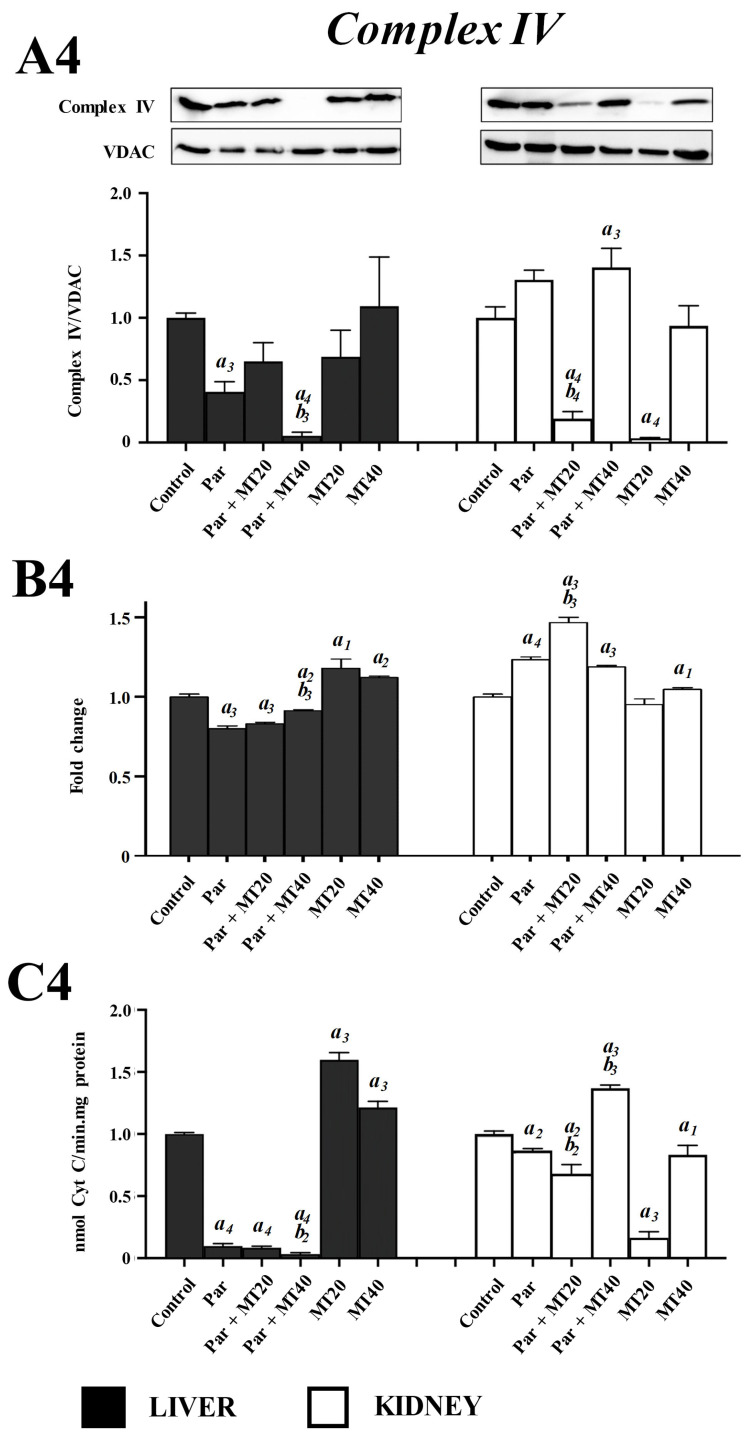
Effects of paracetamol and mitotempo on mitochondrial electron transport chain (ETC) complexes in liver and kidney tissues. The ETC complex number is shown at the top center of each panel. (**A1**–**A4**) Protein abundance of ETC complexes was assessed by Western blotting. (**B1**–**B4**) mRNA expression levels of representative ETC complex subunits were quantified by real-time PCR. (**C1**–**C4**) Enzymatic activities of ETC complexes were determined by photometric assays in isolated liver and kidney mitochondria. Data are presented as mean ± SEM. *a*, vs. control; *b*, vs. paracetamol. Numbers 1–4 indicate significance levels of *p* < 0.05, *p* < 0.01, *p* < 0.001, and *p* < 0.0001, respectively. For each ETC complex, protein abundance, mRNA expression, and enzymatic activity were analyzed independently using one-way ANOVA followed by Dunnett’s multiple comparisons test. Effect sizes are reported as R^2^ values, reflecting the proportion of variance explained by treatment (R^2^ range: 0.85–0.99).

In liver mitochondria, Complex I protein abundance was significantly reduced in the PAR, PAR + MT20, and MT20 groups compared with controls, whereas a significant increase was observed in the MT40 group (Figure 4). Relative to PAR alone, a modest but significant increase in Complex I protein levels was detected only in the PAR + MT20 group. In kidney mitochondria, Complex I protein levels were significantly increased in the PAR, PAR + MT40, and MT40 groups compared with controls, while a significant decrease was observed in the MT20 group. Compared with PAR alone, Complex I protein abundance was significantly reduced only in the PAR + MT20 group. Analysis of mRNA expression revealed that, in liver tissue, Complex I transcript levels were significantly decreased in the PAR and PAR + MT20 groups, whereas significant increases were observed in the PAR + MT40, MT20, and MT40 groups compared with controls. Relative to PAR alone, Complex I mRNA expression was significantly increased in both PAR + MT20 and PAR + MT40 groups. In kidney tissue, Complex I mRNA levels were significantly increased in all treatment groups except MT20 compared with controls. However, relative to PAR alone, a significant reduction in mRNA expression was detected only in the PAR + MT40 group. Complex I enzymatic activity in liver mitochondria was not significantly altered in any treatment group. In contrast, in kidney mitochondria, PAR treatment significantly increased Complex I activity compared with controls, whereas significant reductions were observed in the PAR + MT20, MT20, and MT40 groups. Relative to PAR alone, Complex I activity was significantly decreased in both PAR + MT20 and PAR + MT40 groups (Figure 4).

#### 3.4.2. Complex II

In liver mitochondria, Complex II protein levels were significantly reduced in the PAR, PAR + MT20, PAR + MT40, and MT20 groups compared with controls, whereas MT40 treatment resulted in a significant increase. Compared with PAR alone, Complex II protein abundance was significantly reduced in both PAR + MT20 and PAR + MT40 groups. In kidney mitochondria, a significant increase in Complex II protein levels was observed only in the PAR group, while significant decreases were detected in the PAR + MT20, MT20, and MT40 groups. Relative to PAR alone, a significant reduction was observed exclusively in the PAR + MT20 group. In liver tissue, Complex II mRNA expression was significantly increased in the PAR group but significantly decreased in the PAR + MT20, PAR + MT40, and MT40 groups compared with controls. Compared with PAR alone, significant reductions were observed in both PAR + MT20 and PAR + MT40 groups. In kidney tissue, Complex II mRNA levels were significantly increased in the PAR, PAR + MT20, and PAR + MT40 groups compared with controls. Relative to PAR alone, a significant decrease was detected both in the PAR + MT20 and PAR + MT40 groups. Complex II enzymatic activity was not significantly altered in liver mitochondria. In kidney mitochondria, PAR treatment significantly reduced Complex II activity compared with controls, whereas PAR + MT20 co-treatment significantly increased activity relative to PAR alone (Figure 4).

#### 3.4.3. Complex III

In liver mitochondria, Complex III protein levels were significantly reduced in the PAR and PAR + MT40 groups compared with controls. Relative to PAR alone, a significant decrease was observed only in the PAR + MT40 group. In kidney mitochondria, PAR treatment resulted in a significant increase in Complex III protein levels compared with controls, whereas significant decreases were observed in the PAR + MT20, MT20, and MT40 groups. Compared with PAR alone, a significant reduction was detected only in the PAR + MT20 group. In liver tissue, Complex III mRNA expression was significantly decreased in the PAR and PAR + MT20 groups but significantly increased in the PAR + MT40, MT20, and MT40 groups compared with controls. Relative to PAR alone, mRNA levels were significantly increased in both PAR + MT20 and PAR + MT40 groups. In kidney tissue, Complex III mRNA expression was significantly increased in the PAR group compared with controls, whereas significant decreases were observed in the PAR + MT20 and PAR + MT40 groups relative to PAR alone. Complex III enzymatic activity in liver mitochondria was significantly reduced in the PAR, PAR + MT20, PAR + MT40, and MT20 groups compared with controls. In kidney mitochondria, activity was significantly increased in the PAR and MT40 groups, whereas significant decreases were observed in the PAR + MT20 and MT20 groups. Relative to PAR alone, Complex III activity was significantly reduced in both PAR + MT20 and PAR + MT40 groups.

#### 3.4.4. Complex IV

In liver mitochondria, Complex IV protein levels were significantly reduced in the PAR and PAR + MT40 groups compared with controls (Figure 4). In kidney mitochondria, Complex IV protein abundance was significantly increased in the PAR + MT40 group, whereas significant decreases were observed in the PAR + MT20 and MT20 groups. Relative to PAR alone, a significant reduction was detected only in the PAR + MT20 group. Analysis of mRNA expression showed that, in liver tissue, Complex IV transcript levels were significantly increased in the MT20 and MT40 groups but significantly decreased in the PAR, PAR + MT20, and PAR + MT40 groups compared with controls. Compared with PAR alone, significant but marginal increases were observed in both PAR + MT20 and PAR + MT40 groups. In kidney tissue, Complex IV mRNA levels were significantly increased in the PAR, PAR + MT20, PAR + MT40 and MT40 groups compared with controls, with an additional significant increase in the PAR + MT20 group relative to PAR alone. Complex IV enzymatic activity in liver mitochondria was significantly reduced in the PAR, PAR + MT20, and PAR + MT40 groups but significantly increased in the MT20 and MT40 groups compared with controls. Relative to PAR alone, a significant decrease was observed only in the PAR + MT40 group. In kidney mitochondria, Complex IV activity was significantly increased in the PAR + MT40 group, whereas significant decreases were observed in all other treatment groups. Compared with PAR alone, significant reductions were detected in both PAR + MT20 and PAR + MT40 groups (Figure 4).

## 4. Discussion

PAR-induced toxicity is classically associated with mitochondrial dysfunction driven by oxidative and nitrosative stress, particularly in the liver [1,2,3,4,20,27]. Although mitochondrial oxidative stress is also implicated in PAR-induced nephrotoxicity, its mechanistic contribution in the kidney remains less clearly defined [10,11,12]. The present study provides a comprehensive, organ-specific evaluation of mitochondrial redox status, bioenergetic function, and ETC integrity in response to PAR intoxication and modulation by the mitochondria-targeted antioxidant MT. Our findings demonstrate that MT exerts dual, dose- and organ-specific effects, conferring partial protection in some settings while exacerbating mitochondrial dysfunction in others. Consistent with previous reports, PAR administration resulted in pronounced hepatocellular injury, reflected by marked increases in serum ALT and AST activities, accompanied by mitochondrial oxidative stress, lipid peroxidation, glutathione redox imbalance, oxidative DNA damage, and enhanced mPTP opening in liver mitochondria [1,2,3,4,13,27]. These alterations are in line with the established paradigm in which PAR-derived *N*-acetyl-*p*-benzoquinone imine (NAPQI) disrupts mitochondrial redox homeostasis, leading to impaired oxidative phosphorylation and loss of mitochondrial integrity [1,13,27]. In contrast, renal injury markers were more modest, supporting the concept that PAR-induced nephrotoxicity follows a mechanistically distinct trajectory that is less dependent on canonical mitochondrial death signaling pathways [10,11,12]. A key finding of this study is that MT did not uniformly protect against PAR-induced mitochondrial damage. While higher-dose MT attenuated several indices of mitochondrial oxidative stress and partially restored mitochondrial membrane potential and ETC function in the liver, lower-dose MT and MT administered alone frequently failed to confer protection and, in some cases, exacerbated oxidative stress parameters. These observations align with accumulating evidence indicating that mitochondria-targeted antioxidants exert context-dependent effects on mitochondrial bioenergetics and redox balance, extending beyond simple radical scavenging. Indeed, compounds such as MitoQ have been shown to alter mitochondrial membrane lipid composition, including cardiolipin content, with downstream consequences for ETC organization and function [13,14,15]. Similar mechanisms may underlie the divergent effects observed with MT in the present study. The organ-specific responses to MT were particularly evident in the kidney. Unlike the liver, kidney mitochondria exhibited limited protection by MT against PAR-induced oxidative stress and ETC disruption, and MT alone often induced adverse changes in redox balance, mPTP opening, and mitochondrial membrane potential. This finding is mechanistically important because renal cells lack robust mitochondrial death signaling pathways analogous to those described in hepatocytes [1,2,3,4,10,12]. Therefore, mitochondrial oxidative stress in the kidney is more likely to contribute to dysfunction through non-lethal but bioenergetically compromising mechanisms. Our data therefore support the notion that therapeutic strategies effective in mitigating hepatic mitochondrial injury cannot be assumed to translate directly to renal protection. Analysis of ETC complexes I–IV further underscores the complexity of MT action. PAR-induced disruptions at the mRNA, protein, and activity levels were only partially reversed by MT, and the direction and magnitude of these effects varied markedly between liver and kidney tissues. Notably, restoration of ETC protein expression did not always correspond to recovery of enzymatic activity. This discrepancy suggests potential alterations in supercomplex assembly [28], post-translational modifications [29], or mitochondrial membrane architecture [13,14,15]. In line with this interpretation, recent studies have emphasized that mitochondrial function in metabolically active tissues is governed by tightly regulated, tissue-specific coordination between nuclear- and mitochondrial-encoded OXPHOS genes rather than by protein abundance alone. Organ-specific differences in mitochondrial activity, substrate utilization, and supercomplex plasticity have been reported even in large animal models. These findings underscore that OXPHOS regulation is highly context-dependent and sensitive to metabolic demand and the redox environment. Such findings support our observation that restoration of ETC protein levels does not necessarily predict functional recovery, particularly when mitochondrial membrane organization and post-translational regulation are altered in a tissue-specific manner [30]. These findings emphasize that assessment of mitochondrial function based solely on antioxidant capacity or single endpoints may be insufficient to predict functional outcomes. A limitation of the present study is that the mechanistic basis underlying the observed organ- and dose-specific differences in MT effects was not directly examined. Specifically, potential alterations in mitochondrial supercomplex assembly, post-translational modifications, or membrane organization were inferred from discrepancies between protein expression and enzymatic activity but were not experimentally validated. Moreover, the analyses were limited to liver and kidney tissues; therefore, it remains unclear whether similar MT-related effects occur systemically in other organs.

## 5. Conclusions

Collectively, the present study highlights several important implications. First, mitochondria-targeted antioxidants such as MT exert pleiotropic effects on mitochondrial structure, redox balance, and bioenergetics that are highly dependent on dose and tissue context. Second, mitochondrial oxidative stress plays distinct roles in PAR-induced hepatotoxicity and nephrotoxicity, necessitating organ-specific evaluation of therapeutic interventions. Finally, our findings caution against the uncritical use of mitochondria-targeted antioxidants as broadly protective agents in drug-induced organ toxicity.

In conclusion, while MT can modulate PAR-induced mitochondrial dysfunction, its effects are neither uniformly protective nor predictable across organs. These results underscore the need for careful dose optimization and organ-specific assessment when considering mitochondria-targeted antioxidants as therapeutic strategies for drug-induced toxicity and highlight the importance of comprehensive mitochondrial functional analyses in preclinical safety evaluations.

## Figures and Tables

**Table 1:** Sequences of the primers used for quantitative real-time PCR analysis of mitochondrial ETC components.

Gene Name	Forward (5′→3′)	Reverse (5′→3′)
Complex I	5′-GCTTTAACGAGCCGTAGCCCA-3′	5′-GGGTCAGGCTGGCAGAAGTAA-3′
Complex II	5′-GCTCGAGCTCTCCTACTCC-3′	5′-GCTTGGTGACAGGTGAATGT-3′
Complex III	5′-TCCTTCATGTCGGACGAGGC-3′	5′-AATGCTGTGGCTATGACTGCG-3′
Complex IV	5′-ACCTGGTGAACTACGACTGCT-3′	5′-TCCTAGGGAGGGGACTGCTC-3′
16SRNA	5′-ACACCGGAATGCCTAAAGGA-3′	5′-ATACCGCGGCCGTTAAACTT-3′

## Data Availability

The raw images of Western blots were submitted to the journal for review, but not for publication. All other details can be provided by the corresponding author upon reasonable request.

## Supplementary Materials

The following supporting information can be downloaded at: https://www.mdpi.com/article/10.3390/biom16040556/s1, Figure S1. Raw western blot images of mitochondrial Complex I protein in liver. Figure S2. Raw western blot images of mitochondrial Complex I protein in kidney. Figure S3. Raw western blot images of mitochondrial Complex II protein in liver. Figure S4. Raw western blot images of mitochondrial Complex II protein in kidney. Figure S5. Raw western blot images of mitochondrial Complex III protein in liver. Figure S6. Raw western blot images of mitochondrial Complex III protein in kidney. Figure S7. Raw western blot images of mitochondrial Complex IV protein in liver. Figure S8. Raw western blot images of mitochondrial Complex IV protein in kidney. Figure S9. Raw western blot images of mitochondrial VDAC expression in liver. Figure S10. Raw western blot images of mitochondrial VDAC expression in kidney.

## Abbreviations

The following abbreviations are used in this manuscript:

AIF	Apoptosis-inducing factor
ALT	Alanine aminotransferase
AST	Aspartate aminotransferase
ALP	Alkaline phosphatase
BUN	Blood urea nitrogen
cDNA	Complementary DNA
ETC	Electron transport chain
8-OHdG	8-Hydroxy-2′-deoxyguanosine
JNK	c-Jun N-terminal kinase
mPTP	Mitochondrial permeability transition pore
MT	Mitotempo
NAC	*N*-acetylcysteine
NAPQI	*N*-acetyl-*p*-benzoquinoneimine
PAR	Paracetamol
ROS	Reactive oxygen species
SOD2	Mitochondrial superoxide dismutase
