# Supplementary material for: Dose- and Organ-Specific Dual Effects of MitoTempo in Paracetamol-Induced Hepatorenal Toxicity in Mice
Source: Biomolecules. 2026 Apr 9;16(4):556. doi: 10.3390/biom16040556 (PMC13114090; doi:10.3390/biom16040556)
Supplement: Supplementary file 1 [file biomolecules-16-00556-s001.zip › biomolecules-4100534-supplementary.pdf]

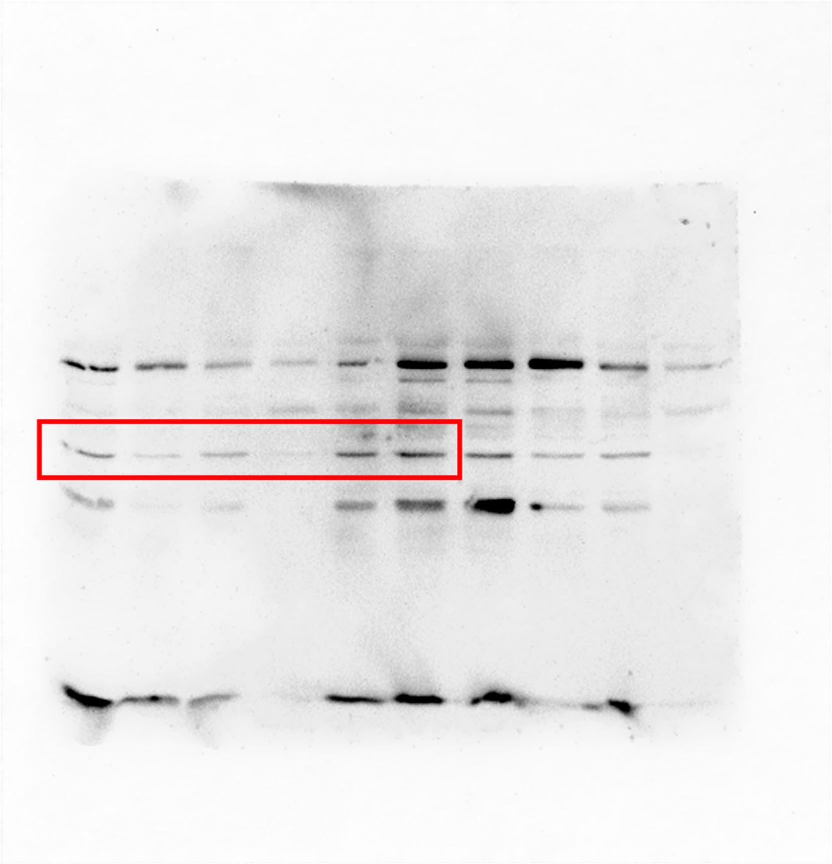

Figure S1. Raw western blot images of mitochondrial Complex I protein in liver.

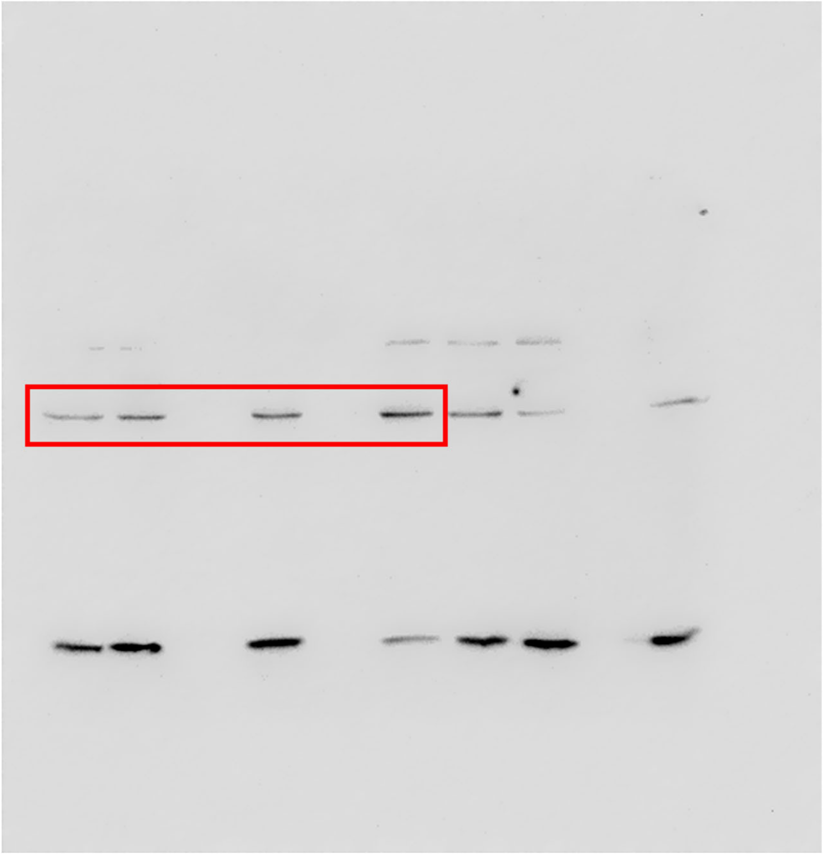

Figure S2. Raw western blot images of mitochondrial Complex I protein in kidney.

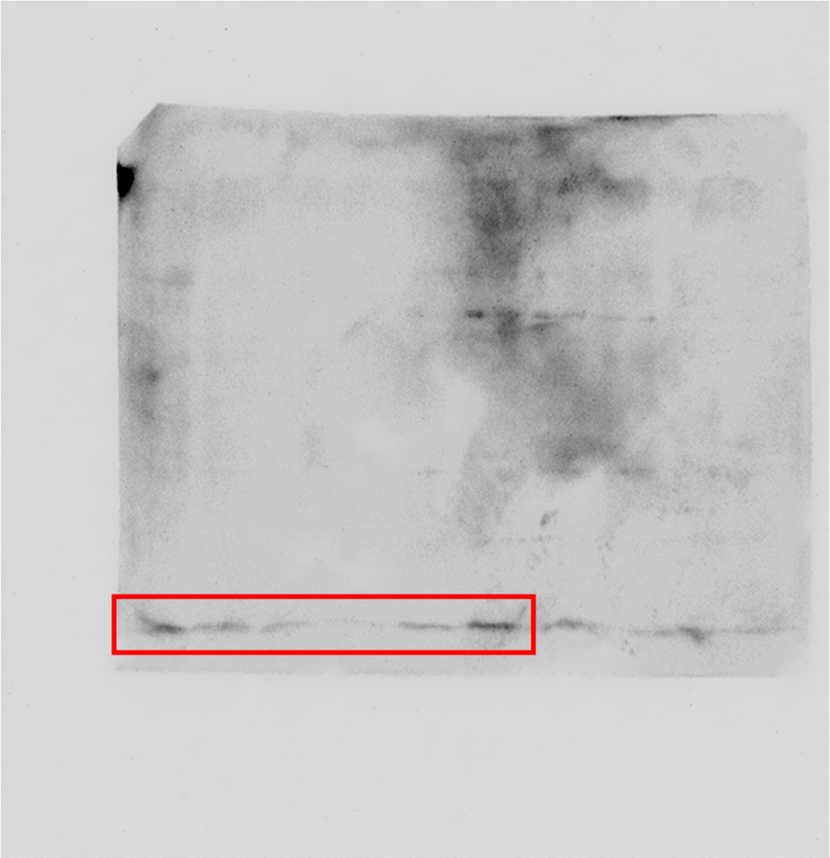

Figure S3. Raw western blot images of mitochondrial Complex II protein in liver.

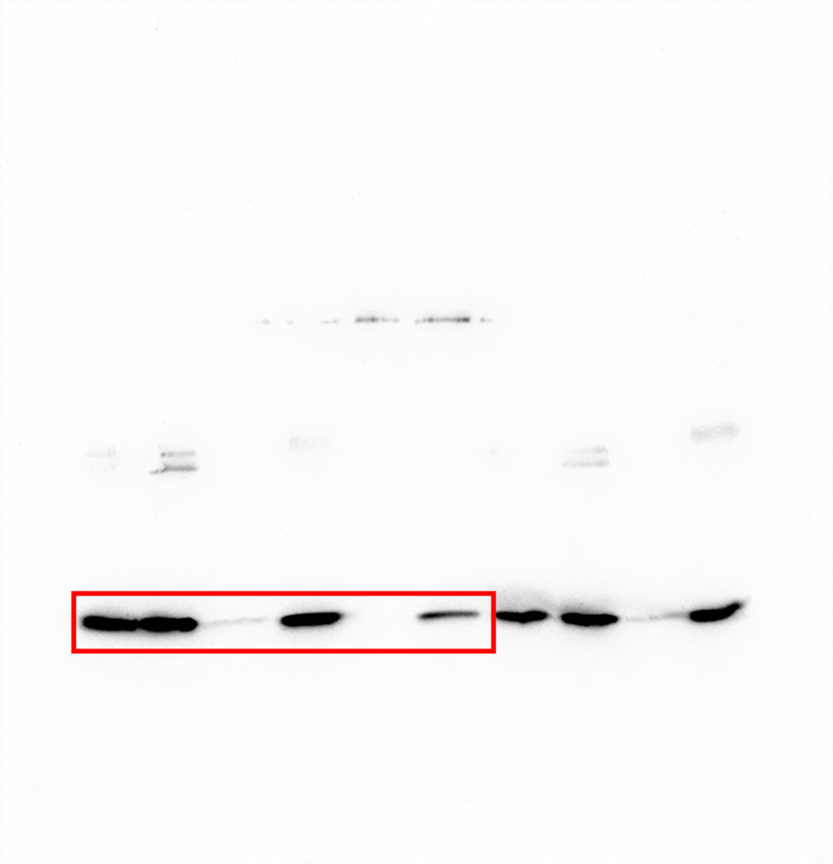

Figure S4. Raw western blot images of mitochondrial Complex II protein in kidney.

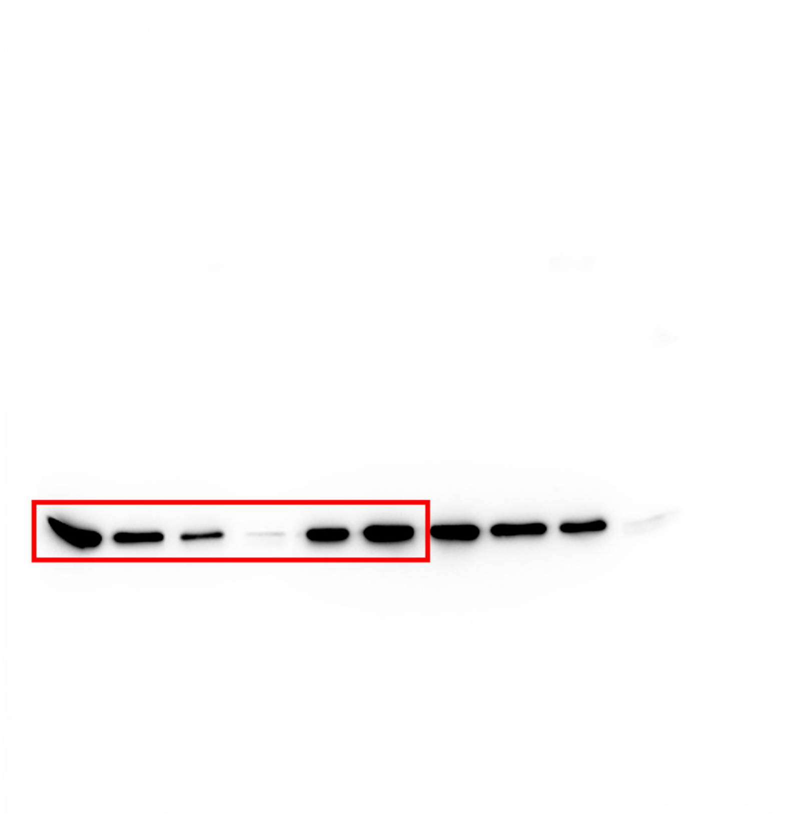

Figure S5. Raw western blot images of mitochondrial Complex III protein in liver.

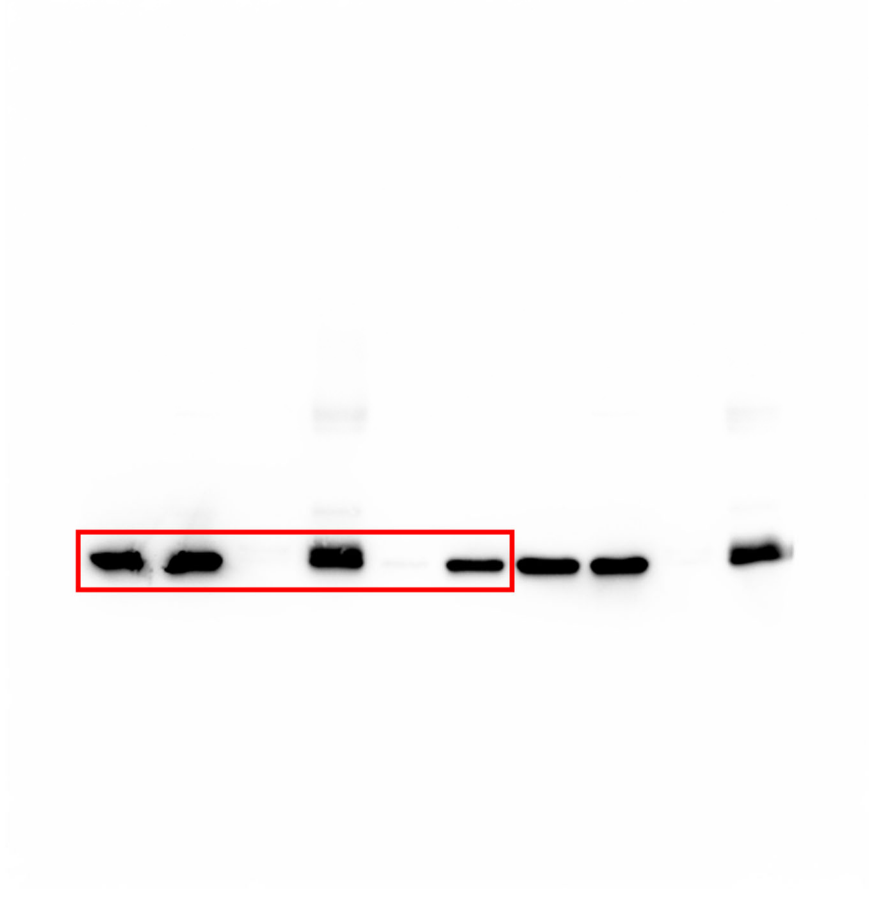

Figure S6. Raw western blot images of mitochondrial Complex III protein in kidney.

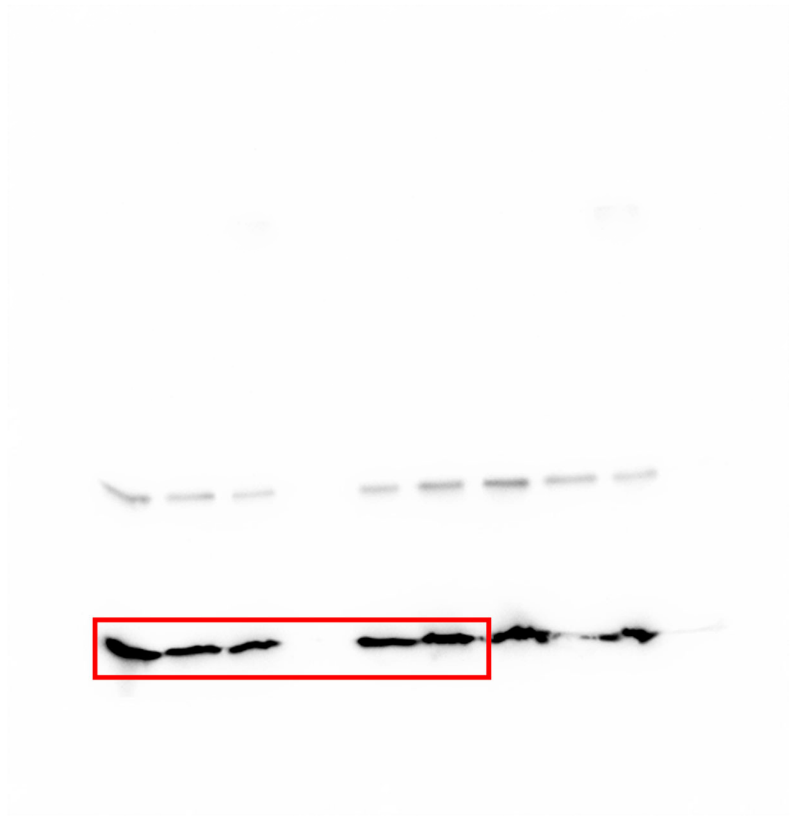

Figure S7. Raw western blot images of mitochondrial Complex IV protein in liver.

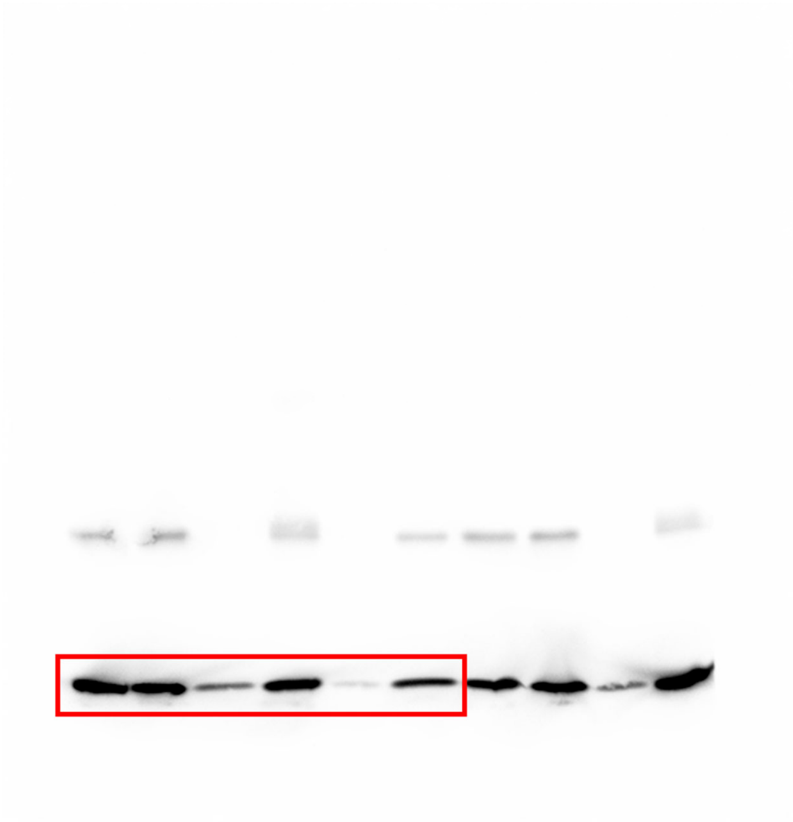

Figure S8. Raw western blot images of mitochondrial Complex IV protein in kidney.

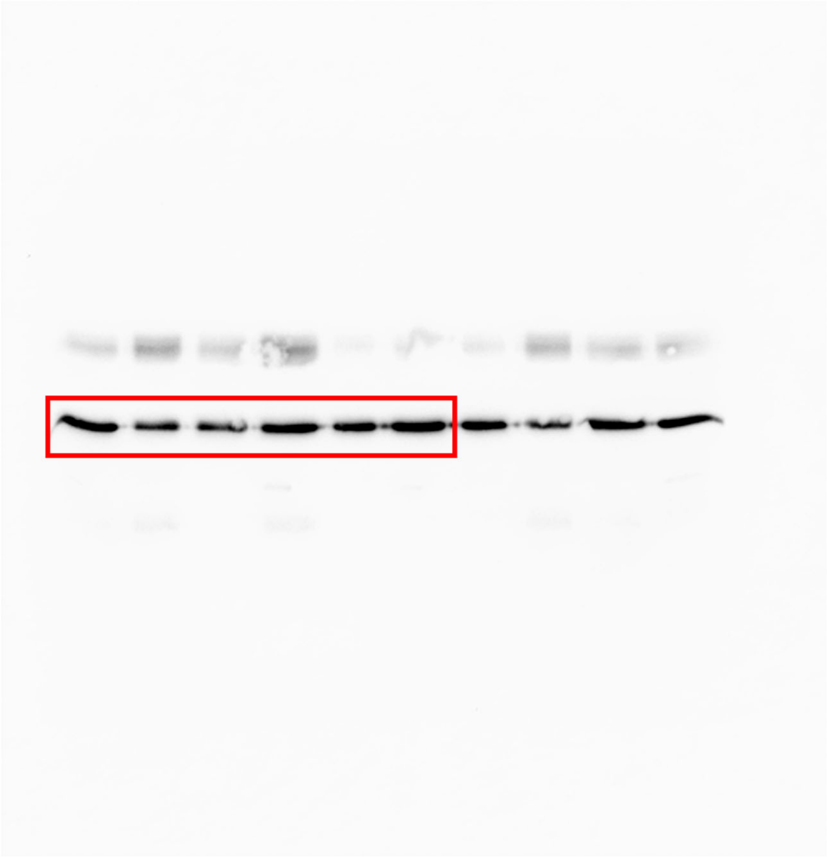

Figure S9. Raw western blot images of mitochondrial VDAC expression in liver.

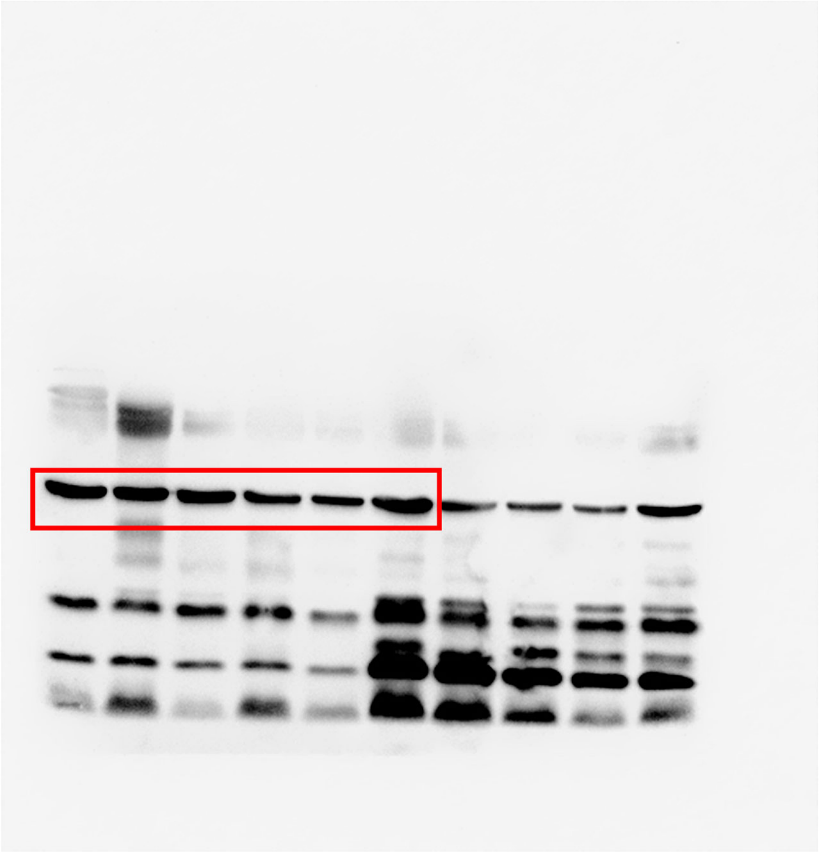

Figure S10. Raw western blot images of mitochondrial VDAC expression in kidney.
